# Protein-Based Nanohydrogels for Bioactive Delivery

**DOI:** 10.3389/fchem.2021.573748

**Published:** 2021-07-09

**Authors:** Subhash Chander, Giriraj T. Kulkarni, Neerupma Dhiman, Harsha Kharkwal

**Affiliations:** ^1^ Amity Institute of Phytochemistry and Phytomedicine, Amity University, Noida, India; ^2^ Amity Institute of Pharmacy, Amity University, Noida, India; ^3^ Gokaraju Rangaraju College of Pharmacy, Hyderabad, India

**Keywords:** nanohydrogels, natural protein, biodegradability, drug delivery, self-assembly, polymeric networks

## Abstract

Hydrogels possess a unique three-dimensional, cross-linked network of polymers capable of absorbing large amounts of water and biological fluids without dissolving. Nanohydrogels (NGs) or nanogels are composed of diverse types of polymers of synthetic or natural origin. Their combination is bound by a chemical covalent bond or is physically cross-linked with non-covalent bonds like electrostatic interactions, hydrophobic interactions, and hydrogen bonding. Its remarkable ability to absorb water or other fluids is mainly attributed to hydrophilic groups like hydroxyl, amide, and sulphate, etc. Natural biomolecules such as protein- or peptide-based nanohydrogels are an important category of hydrogels which possess high biocompatibility and metabolic degradability. The preparation of protein nanohydrogels and the subsequent encapsulation process generally involve use of environment friendly solvents and can be fabricated using different proteins, such as fibroins, albumin, collagen, elastin, gelatin, and lipoprotein, etc. involving emulsion, electrospray, and desolvation methods to name a few. Nanohydrogels are excellent biomaterials with broad applications in the areas of regenerative medicine, tissue engineering, and drug delivery due to certain advantages like biodegradability, biocompatibility, tunable mechanical strength, molecular binding abilities, and customizable responses to certain stimuli like ionic concentration, pH, and temperature. The present review aims to provide an insightful analysis of protein/peptide nanohydrogels including their preparation, biophysiochemical aspects, and applications in diverse disciplines like in drug delivery, immunotherapy, intracellular delivery, nutraceutical delivery, cell adhesion, and wound dressing. Naturally occurring structural proteins that are being explored in protein nanohydrogels, along with their unique properties, are also discussed briefly. Further, the review also covers the advantages, limitations, overview of clinical potential, toxicity aspects, stability issues, and future perspectives of protein nanohydrogels.

## Introduction

Gels are considered an intermediate between the liquid and solid states which lack a flow property. The gel-based delivery vehicle can be classified into three main categories: organogel, hydrogel, and biogel, depending upon the type of medium it absorbs like organic material, water, or both, respectively (Iemma et al., 2006). Hydrogels possess unique three-dimensional polymeric networks that can absorb significant amounts of water including biological fluids without dissolving themselves.

Hydrogels can be fabricated into different physical forms like microparticles, nanoparticles, films, and slabs (Hoare and Kohane, 2008). Biopolymeric hydrogels have also been used as a carrier for topical and localized drug delivery due to their tunable viscoelasticity, excellent water absorption ability, and biocompatibility. The polymeric materials used in hydrogels are obtained from natural as well as synthetic resources. Synthetic hydrogels are associated with issues related to their biocompatibility and biodegradability. However, such hydrogels possess good mechanical strength and water absorption capacities (Sosnik and Seremeta, 2017; Chyzy et al., 2020; Shen et al., 2011).

### Differences Between Nanohydrogels and Conventional Hydrogels

Nanohydrogels possess the unique properties of the hydrogel as well as nanoparticles, with sizes ranging from 1 to 100 nm. Conventional hydrogels like macro or microgels generally involve intermolecular crosslinking while in nanohydrogels, intramolecular crosslinking is predominantly identified (Dalwadi and Patel, 2015). Nanohydrogels can better retain incorporated drugs due to their small size compared to macro or micro hydrogels with comparatively larger sizes (Schwall and Banerjee, 2009). Nanohydrogels are being investigated in the delivery of bioactive compounds at a rapid pace due to several advantages over conventional hydrogels like longer half-life in plasma, better loading capacity, and superior tissue uptake (Gao et al., 2008; Fang et al., 2011; Sharpe et al., 2014; Chyzy et al., 2020).

Moreover, nanohydrogels possess better suitability for the parenteral route of administration, as these can move inside the fine capillaries due to their small size. So, all these factors make nanohydrogels a better and preferred choice for the delivery of bioactive compounds over conventional hydrogel systems (Dalwadi and Patel, 2015; Chacko et al., 2012; Hamidi et al., 2008; Ferreira et al., 2013; Buwalda et al., 2017).

### Advantages of Protein Nanohydrogels Over Polysaccharide-Based Hydrogels

Protein/peptide and polysaccharide-derived natural hydrogels are biodegradable with excellent biocompatibility. Moreover, such hydrogels also exhibit tunable mechanical properties and responses to specific stimuli such as ionic concentration, temperature, and pH, etc., which have enhanced the interest of researchers in different fields like tissue engineering, regenerative medicine, and delivery of bioactive compounds (Rambhia and Ma, 2015).

Availability of more functional groups for modification (thiol, amino, hydroxyl, and carboxyl), more sensitive delivery toward the external stimuli, special recognition ability of some peptides, and self-assembling ability of certain peptides/proteins make these nanohydrogels superior when compared to polysaccharide-based nanohydrogels (Ren et al., 2017; Zhang et al., 2013; Cai et al., 2017).

Additionally, one of the most common challenges associated with natural polysaccharide-based hydrogels is the wide variability in their molecular weight which requires advanced purification and sophisticated analytical technologies for assessment (Wang et al., 2020). On the other hand, proteins or peptides generally possess a definite amino acids sequence, fixed molecular weight, uniform physicochemical properties, and show less batch to batch variation. Protein/peptide-based nanohydrogels can be utilized for the encapsulation of hydrophilic as well as hydrophobic drugs by selecting a suitable protein/peptide constituent. Protein, as the macromolecule, can easily encapsulate the other macromolecules for delivery like proteins and nucleic acid (DNA/RNA) (Wang Q. et al., 2019). Physical properties of the proteins like solubility, folding, de-folding, etc. are highly dependent upon environmental factors like pH, ionic concentration, and temperature, for example, the properties of the peptides/proteins significantly change at the isoelectric point which is utilized for the responsive sensitive delivery of bioactive compounds (Cheng R. et al., 2013; Rosa et al., 2020).

### Discussion on Other Nanocarrier Delivery Systems

#### Nanoparticles (NPs)

Polymeric and lipid-based nanoparticles have diverse biomedical applications in drug delivery, diagnostics, and tissue engineering (Calzoni et al., 2019; Khan et al., 2019). These nanoparticles have certain limitations, for example, the use of solid lipid nanoparticles as a delivery system exhibits drug burst release, low payload capacity—especially for hydrophilic drugs, and accumulation in the liver and spleen, etc. (Ghasemiyeh and Mohammadi-Samani, 2018). Polymeric nanoparticles comprise both natural as well as synthetic polymers. Limitations pertaining to biocompatibility and undesired immunological reactions make synthetic polymers less desirable than natural polymers (Ghasemiyeh and Mohammadi-Samani, 2018; Khan et al., 2019). NPs formulated using polylactic acid (PLA), a copolymer with glycolic acid (PLGA), and polyethylene glycol (PEG) are frequently reported for delivery of bioactive compounds. Although, these NPs generally produce non-toxic monomers or oligomers inside the body, but in certain instances, reagents and surfactants used during the preparation or functionalization remain in traces leading to toxicity (Calzoni et al., 2019).

#### Liposomes

Liposomes are generally nanosized spherical vesicular structures consisting of an aqueous core surrounded by the lipid bilayer (phospholipid layers) These are well explored for the delivery of nutrients and other bioactive compounds due to their good compatibility, degradability, and flexible physicochemical properties (Sercombe et al., 2015).

Despite several advantages, rapid clearance by the reticuloendothelial system (RES) remains one of the foremost challenges for this formulation. Another significant limitation of liposomes is opsonization, a process in which several serum proteins get adhered to their surface which speeds up its clearance. To slow down or manage opsonization, liposomes are generally coated with poly(ethylene glycol) (PEG) ultimately enhancing its circulation in plasma (Nosova et al., 2019).

Furthermore, repeated administration of PEGylated liposomes again significantly decreases its plasma half-life, due to the phenomenon commonly known as “accelerated blood clearance” (ABC) (Dams et al., 2000). Innate immune responses have been reported several times upon the administration of liposomes, which in turn triggers an acute hypersensitivity syndrome (La-Beck et al., 2019).

#### Micelles

Polymeric micelles are nanosized particles consisting of amphiphilic block copolymers as monomer units. These are more frequently reported for the delivery of hydrophobic drugs and have recently attracted attention as novel drug vehicles due to stability, biocompatibility, and loading ability. The common challenge associated with *in vivo* use of micelles is their dissociation into monomeric units. This process gets hastened upon binding of monomeric units which in turn binds with other blood components such as proteins (Yokoyama, 2014; Ahmad et al., 2014).

#### Nanocapsules

Nanocapsules are colloidal drug delivery systems of submicron size comprising a hollow core surrounded by a thin membrane of natural or synthetic polymers. They generally possess spherical shapes with a size in the range of 50–300 nm. Nanocapsules have attracted the substantial interest of researchers due to several merits, namely the ability to carry biomolecules as well as small molecules of a wide polarity range, high loading capacity, and control release ability. Research revealed that nanocapsules can protect highly unstable bioactive compounds and some reports also support their site-specific drug delivery ability (Mora-Huertas et al., 2010; Verma et al., 2017). Despite several advantages, there are some challenges associated with nanocapsules such as stability issues and costly production at large scale because of sophisticated preparation techniques (Patra et al., 2018).

### Classification, Preparation, and Structural Modification of Protein Nanohydrogels

Hydrogels can be classified based upon various criteria, i.e., the nature of building blocks, origin of the polymeric system, and its method of preparation/crosslinking. Based upon the nature of the building blocks, these are broadly classified as ionic or neutral hydrogels. Hydrogels have been classified as synthetic, natural, or hybrids depending on the origin of these polymeric systems. The nature of the crosslinking of these hydrogels leads to categorization into chemical, physical, and biochemical or enzymatic hydrogels (Sosnik and Seremeta, 2017; Panahi and Baghban-Salehi, 2019).

Preparation of protein nanohydrogels is mainly reported by either using a physical method or a chemical method of crosslinking (Wang Q. et al., 2019; Mondal et al., 2020).

Physically cross-linked protein hydrogels are stabilized by various non-covalent bonding interactions such as ionic, hydrogen bonding, van der Waal, and hydrophobic interactions. Such nanohydrogels are less stable; and changes in the physical environment like pH, temperature, and concentration of ions or another solute, can easily disturb the 3D hydrogel network (Yang et al., 2016; Mondal et al., 2020).

Hydrogels prepared by the physical crosslinking method exhibit limited volume change during conversion from a solution to a gel state. Such gels generally remain as low viscous solutions. A physically cross-linked hydrogel is found to be more sensitive to mechanical forces and exhibits a weaker gel property in comparison to the chemical cross-linked hydrogel. In general, physically cross-linked hydrogels remain devoid of organic solvents and other coupling reagents, which make them more suitable for drug delivery applications including injectable use (Nguyen and Lee, 2010).

Hydrogels prepared by the chemical methods usually involve covalent bonds which make them stable toward environmental conditions. Hydrogels prepared by chemical reactions undergo a significant change in volume when converted from the solution state to gel. Common strategies of crosslinking are disulfide bond formation between thiol groups, light-induced polymerization, and specific reactions of functional groups like amine, carboxylic acid with aldehyde, sulfones, or acrylates (Nguyen and Lee, 2010; Mondal et al., 2020).

In many instances, natural proteins are conjugated with synthetic polymers to prepare hydrogels of improved mechanical strength, such hydrogels are known as hybrid-hydrogels (Stevens et al., 2012; Panahi and Baghban-Salehi, 2019). Conjugation of proteins with other polymers depend upon a number of factors such as the presence of accessible reactive groups, reaction conditions, and solubility (Matsumoto et al., 2013; Mondal et al., 2020; Sletten and Bertozzi, 2009). Amino acids like lysine and cysteine easily form covalent attachments (Sletten and Bertozzi, 2009). Proteins with a limited number of free amino acids cannot be easily linked with other moieties. In such cases, the additional number of amino acids can be added with the help of genetic engineering (Bontempo et al., 2004; Rana et al., 2010).

Functional properties of proteins can be enhanced or altered to perform a specific function via attachment with certain moieties like DNA or another peptide via genetic engineering. Such DNA-protein conjugates are reported for a broad range of applications including the delivery of bioactive macromolecule like nucleic acid and enzymes (Zhao et al., 2010; Diaz et al., 2018; Liu et al., 2017).

Proteins possess several reactive functional groups in their side chains apart from the terminal amino and carboxylate groups which are utilized for bioconjugation using various types of specific crosslinking agents, for example, the free amino of lysine, thiol of cysteine, and the carboxylate of glutamate or aspartate are commonly coupled with N-hydroxy-succinimides, maleimides, and carbodiimides, respectively (Cortajarena and Grove, 2016; Diaz-Perlas et al., 2018). Apart from small molecules, additional materials like polymers and nanoparticles can be attached to proteins for the development of drug delivery systems (Grover and Maynard, 2010).

### Characterization of Protein-Based Hydrogels

Protein-based nanohydrogels are characterized by various tests on the basis of their structure and area of application (Table 1). Characterization can be broadly categorized into different categories like; rheology, morphology, chemical functionality, and thermal stability (Rahman et al., 2019).

**TABLE 1:** General characterization of protein-based nanohydrogels.

Type of characterization	Rheological characterization	Chemical functionality	Morphology and porosity	Thermal stability
Parameters/properties	Viscoelastic character and other relevant parameters like gel strength, gelation time, and yield-strain	Molecular structure and elemental composition	Shape, surface morphology, pore size, pore size distribution, and information of inside the matrix and its nanomaterial	Change in structural strength, physical state, glass transition temperature, and melting point
Techniques/instruments	Strain sweep test, frequency sweep test, time sweep test, temperature sweep test	FT-IR	SEM, TEM, STEM, LSCM, FESEM, AND	DSC
		NMR		TGA
		UV-visible spectroscopy, EDS		

#### Rheological Characterization

Rheological characterization of protein nanohydrogels provides key information about viscoelastic properties and reveals the effect of environmental factors like temperature, pH, concentrations of ions, and enzymes, etc. on rheological characteristic parameters, i.e., viscoelastic character, gel strength, gelation time, and yield-strain (Panahi and Baghban-Salehi, 2019; Rahman et al., 2019; Cuomo et al., 2019).

Rheological parameters reflect the various properties of the protein nanohydrogels like stiffness, relative liquid-solid properties, gelation kinetics, viscoelastic regions, and relaxation time scale. To study the rheological behavior, oscillation rheology is used instead of conventional constant stress. A common problem associated with constant stress is the Wiesenberger effect and secondly, it may damage the 3D structures of the hydrogel. Sinusoidal shear is applied in oscillation rheology and the resulting stress is measured as a function of time. Reversible interactions can be easily studied using oscillation rheology. Also, it generally does not damage the 3D structure of the protein (Panahi and Baghban-Salehi, 2019; Rahman et al., 2019; Yan and Pochan, 2010).

#### Morphological Tests

The morphological characteristics of the hydrogel depend upon several factors like; method of preparation, compositions, hydrophobic substitutes, and fabrication techniques (Rahman et al., 2019). Morphological parameters like pore size and pore size distribution are crucial from an application point of view which directly affects the loading as well as the release rate of the bioactive compounds inside the hydrogel matrix. Hydrogel porosity, three-dimensional network, and surface morphology are studied using scanning electron microscopy (SEM). SEM evaporates the water content of the hydrogel which affects the actual characterization. This problem can be resolved using cryo-SEM. SEM is the most commonly used technique for morphological studies of hydrogels regardless of their composition (McMahon et al., 2010; Gunko et al., 2017).

As an alternative to SEM, laser scanning confocal microscopy (LSCM), along with fluorescent dyes, has also been used to study the shape and pore dimensions of hydrogels but this technique is less effective when compared to SEM (Fergg et al., 2001). A field emission scanning electron microscope (FESEM) also provides key information about the morphology of protein nanohydrogels with higher resolution. Moreover, FESEM is also used to determine the elemental composition of protein nanohydrogels (Reichelt et al., 2004).

Other techniques like transmission electron microscopy (TEM) and scanning transmission electron microscopy (STEM) provide the image of the nanohydrogel. Both of these techniques require extra precision in sample preparation and both of them are less informative when compared to SEM (Yan et al., 2006). Atomic force microscopy (AFM) is a newer technique commonly used to study the characterization of polymers including nanohydrogels. This technique does not require electron beam or staining, so without damaging the surface it can provide information on surface morphology as well as the distribution of the nanomaterial inside the hydrogel matrix (Reichelt et al., 2004).

#### Chemical Functionality Test

Spectroscopic techniques like FT-IR and NMR are performed for the investigation of the molecular structure and these constitute qualitative analysis (Yan et al., 2006). The presence of the amide bond in the protein or peptide-based hydrogel can be identified using FT-IR. Release of bioactive compounds with any chromophore can be analyzed using UV-visible spectroscopy and LC-MS (Zheng et al., 2015). Energy dispersive spectroscopy (EDS), also known as energy dispersive X-ray spectroscopy (EDX) is used along with SEM to obtain information about the elemental composition of nanohydrogels. EDS detects emitted X-rays released upon bombarding the sample with an electron beam (Son et al., 2020).

#### Thermal Stability

Change in the structural strength of protein-based nanohydrogels with temperature is measured via a differential scanning calorimeter (DSC) or thermogravimetric analysis (TGA). The relative change in the structure of the hydrogel with temperature is compared with the control state. Parameters like glass transition temperature (Tg) and melting point are important characteristics to reveal the thermoplastic behavior of protein nanohydrogels determined by a DSC or TGA (Wattie et al., 2018; Panahi and Baghban-Salehi, 2019).

### Applications of Protein Nanohydrogels

**FIGURE 1:**
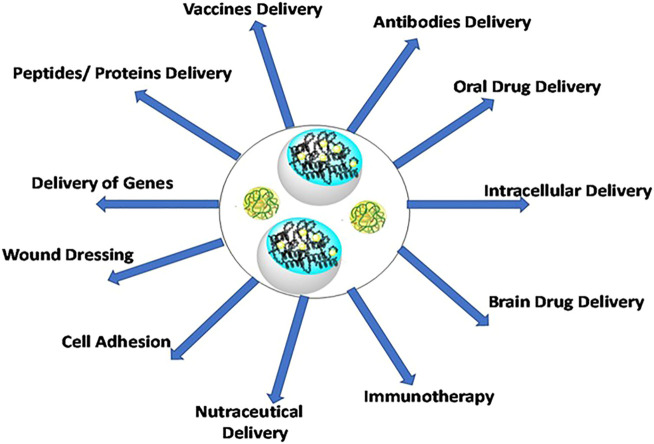
Applications of protein nanohydrogels in delivery of bioactive and biopharmaceutic compounds.

Protein nanohydrogels have applications in diverse disciplines like food industries, tissue engineering, biomedical implants, pharmaceuticals, regenerative medicines, and controlled drug delivery processes (Figure 1) (Soni et al., 2016; Vermonden et al., 2012; Lee et al., 2020; Navarra et al., 2016). Preparation-specific applications of protein nanohydrogels are discussed in “Protein Nanohydrogel reported for specific applications” Section.

## Different Types of Natural Proteins Commonly Used in Nanogels

Proteins of natural origin possess excellent biocompatibility and biodegradability properties which have boosted their demand in bioformulations. Some of the proteins derived from natural sources and frequently utilized in the preparation of protein hydrogels are discussed below.

### Elastin

Elastin is a highly elastic structural protein present in abundance inside the extracellular matrix of connective tissue and is primarily found in organs like the lungs, aorta, and skin. It provides elasticity to the skin around a thousand times more flexible than collagen protein (Matsumoto et al., 2013). Inside the body, elastin is prepared from its precursor tropoelastin, which itself originates from the cells, i.e., smooth muscle cells, fibroblasts, and endothelial cells. Purification of cross-linked elastin is a challenging task because of its insolubility and presence of non-elastin contaminates like tropoelastin. To achieve pure elastin of biological grade, it is prepared over the microfibrillar surface (Mecham, 2008).

Elastin comprises a unique pentapeptide sequence, Valine-Proline-Glycine-X-Glycine, in repetition. In the pentapeptide, the nature of ‘X’ amino acid plays a vital role in deciding the physical properties of the protein, except proline ‘X’ which can be any natural amino acid. Elastin protein is thermal responsive, non-immunogenic, and biocompatible, all such properties make it a suitable candidate for hydrogel preparation (Panahi and Baghban-Salehi, 2019; Jonker et al., 2012).

In a recent study, a biomimetic tendon synthesized using chitosan, poly-ε-caprolactone, and cellulose nanocrystals were coated with elastin, which showed good biocompatibility without immunogenicity (Almeida et al., 2019). Elastin-like proteases (ELPs) are recombinant proteins that contain unique peptide mimics to tropoelastin. ELPs possess biocompatibility like elastin, due to which these have emerged as potentially useful materials for tissue engineering and other biomedical applications (Jeon, 2013; Despanie et al., 2016).

### Collagen

Collagen is an extracellular structural protein, mainly occurring in the connective tissues of a mammal’s body which provides mechanical strength to the body. The total content of the collagen varies from 25 to 35% of the total protein content. Structurally, it occurs in the triple helix, also known as the collagen helix, which contains glycine-proline-hydroxyproline as frequent repeating units (Jia and Kiick, 2009). Collagen is predominantly produced by the fibroblast cells, and it occurs mainly in the ligaments, tendons, and skin. Collagen undergoes mineralization to a different extent. Accordingly, its rigidity also varies in bones, cartilage, and tendons. Collagen is widely used in the field of biomedical engineering, drug delivery, tissue engineering, and recently it has found application for the delivery of proteins, genes, and nucleic acids (Masuda et al., 2004; Khan and Khan, 2013).

The main limitations of collagen are its poor mechanical properties and thrombogenicity. Particularly, due to intrinsic thrombogenicity properties, it activates and adheres the platelets which limit its use for direct systemic administration as well as to the high vascular tissues (Pashneh-Tala et al., 2016; Copes et al., 2019).

Some collagen-based hydrogels commercially available for cell culture are Matrigel^™^, Vitrogen^®^, Collagel^®^, and Thermacol^®^ [Flexcellint website], while another product named Woun'Dres^®^ has been in clinical use for dry wound dressing (Aswathy et al., 2020).

### Gelatin

Gelatin is a colorless, tasteless biopolymer prepared by thermal decomposition of collagen using acids or bases. In the hydrolysis process, the triple-helix of collagen protein breaks into single-strand gelatin molecules. Based upon acidic or basic hydrolysis, gelatin is divided into type A and B, respectively. Basic hydrolysis increases the content of free carboxylic acid by hydrolyzing residues like glutamine and asparagine into glutamic and aspartic acid, respectively. Structurally, gelatin possesses frequent repetition of residues like glycine, 4-hydroxyproline, and proline (Tomka et al., 1975; Bigi et al., 2004). Below 35°C, it turns into a transparent and reversible gel form, which is frequently used in food, pharmaceutical, and cosmetic industries. However, above 35°C, gelatin becomes an individual molecule due to the absence of interchain hydrogen bonding. The gelling properties of gelatin significantly change depending upon the nature of chemical crosslinking. Gelatin films are transparent and flexible with good mechanical strength. Both A and B forms of gelatin have a different affinity toward positively or negatively charged drugs.

Gelatin is a versatile drug delivery carrier in which charged biomolecules can be loaded. Gelatin A and gelatin B have different isoelectric points, moreover, gelatin B contains more free carboxylic acids groups, hence has more binding affinity toward basic drugs compared to gelatin A. Depending on the polarity and electrostatic properties of a drug, a suitable form of gelatin can be selected to maximize drug loading (Santoro et al., 2014).

Gelatin is hydrophilic in nature and due to its excellent film-forming ability, it is commonly used for the construction of capsule shells. It rapidly dissolves in gastric fluids (Kozlov and Burdygina, 1983). However, gelatin possesses poor thermal stability, so in order to improve its stability, it is generally cross-linked with other polymers (Catoira et al., 2019). Certain favorable properties of gelatin such as good biocompatibility, biodegradability, versatility in crosslinking, easy availability, and economic feasibility have led to numerous studies on the preparation of gelatin-based hydrogels as well as their role in bioactive delivery (Rosso and Martinelli, 2014; Santoro et al., 2014; Aswathy et al., 2020; Rosa et al., 2020).

### Silk Fibroin

Silk fibroin (SF) is a water-insoluble protein produced by the larvae of *Bombyx mori* and other moths belonging to genera such as Cricula and Antheraea. Silk mainly consists of three proteins: silk fibroin, core protein, and a glue-like protein, known as sericin. Fibroin consists of repetitive hydrophobic and hydrophilic residues, and in the primary structure, it also possesses frequent repeating units of six amino acids Gly-Ser-Gly-Ala-Gly-Ala. (Hofmann et al., 2006; Gong et al., 2010).

The secondary structure of fibroin protein displays the arrangement of tightly packed antiparallel β-sheets rich in glycine residue (around 45.9%), which may be attributed to the rigid structure of silk with high tensile strength (Vepari and Kaplan, 2007; Gong et al., 2010). These β-sheets of fibroin make them insoluble in water and play a key role in the formation of fibroin hydrogels. Fibroin gelation is found to be enhanced upon increase in concentration as well as rise in temperature. Further, addition of a hydrophilic polymer or increment in the acidity of the medium decreases the hydrophobicity of chains. This consequently enhances the water solubility and ultimately potentiates the gelation process (Kim et al., 2004). Good biodegradability, biocompatibility, and impressive mechanical properties make fibroin an attractive biomaterial for tissue engineering and biomedical use (Altman et al., 2003).

In recent studies, silk fibroin-based hydrogels were reported for different biomedical applications such as bone regeneration, prevention of hypertrophic scars as well as in delivery of bioactive compounds, including insulin (Shi et al., 2017; Song et al., 2020; Maity et al., 2020).

### Globular Proteins

Globular proteins are spherical in shape, possess affinity toward water, and generally form colloids in the aqueous environment. These proteins achieve their spherical shape due to their unique tertiary structure which can be established by dynamic light scattering techniques and ultracentrifugation. In the three-dimensional structure of the globular protein, polar residues remain at the outer vicinity and account for miscibility with water, whereas non-polar residues bind toward the interior.

Globular proteins like β-lactoglobulin, ovalbumin, and bovine serum albumin are commonly used for the preparation of gel. Among these proteins, β-lactoglobulin is predominantly used in the food industry and its behavior is well studied in various scientific reports (Richardson and Ross-Murphy, 1981; Yong and Foegeding, 2010). Other small globular protein known as hen egg-white lysozyme (HEWL) present in the white portion of hen eggs is also explored as a promising biomaterial. In the secondary structure, lysozymes exhibit a combination of α-helices and β-sheets. HEWL is a water-soluble protein, consisting of four disulfide bridges that transform it into gel form via self-assembly.

At neutral pH, HEWL does not undergo gelation even at elevated temperature due to the presence of the stable disulfide bridges. The addition of reducing agents like dithiothreitol (DTT) disrupts the disulfide bridges leading to consequent enhancement in the flexibility of the backbone chain, inducing unfolding and ultimately initiating the self-assembly process to form hydrogel. Heating of HEWL at around 85°C and at neutral pH in the presence of DTT, followed by slow cooling leads to the formation of transparent hydrogels (Yan et al., 2006). Hydrogels based upon globular proteins such as β-lactoglobulin, ovalbumin, and bovine serum albumin are reported for diverse applications in delivery of bioactive large molecules, small drugs, and nutraceuticals (Feng et al., 2016; Shafaei et al., 2017; Panahi and Baghban-Salehi, 2019).

In a recent study reported by Toprakcioglu et al. on nanogels, NGs prepared using β-lactoglobulin, reconstituted silk fibroin, and lysozyme showed remarkable intracellular penetration ability as well as delivery of intracellular cargo (Toprakcioglu et al., 2020)

## Preparation and Applications of Protein-Based Nanohydrogels (NG)

Nanohydrogels comprising of natural biomaterials are commonly prepared from natural proteins/peptides derived from animal or plant resources and polysaccharide polymers such as hyaluronic acid, chitosan, heparin, alginate, and agarose, etc. (Schloss et al., 2016). Certain unique properties of nanogels like rapid swelling-deswelling ability, decent drug loading capacity, less-immunogenicity, and biodegradable behavior, etc. make them a promising biomaterial in drug delivery (Vinogradov, 2010).

Protein nanogels can incorporate hydrophobic as well as hydrophilic drugs. Their extremely small size facilitates easy crossing across the blood-brain barrier (BBB). Nanogels can be prepared at mild conditions, which makes them a suitable vehicle for the encapsulation of thermolabile drugs and other biomacromolecules. The size of the nanogel varies from 20 to 200 nm in diameter, which avoids its uptake into the reticuloendothelial system and renal clearance system (Jonker et al., 2012).

### Studies on Preparation of Protein Nanohydrogels

This section mainly emphasizes the studies reported for the preparation of protein nanohydrogels. In such studies, researchers mainly focused on the preparation and characterization aspects of protein nanogels but not performed applications-based studies using the proposed formulation. This section is further divided into two sub-sections based upon type of method used in the preparation of protein NGs.

#### Protein Nanohydrogels Prepared by Physical Methods

##### Self-Assembling Nanohydrogels of Globular Proteins

Shaoyong et al. prepared nanogels using ovalbumin and lysozyme proteins present in the white portion of a hen egg. To prepare the nanogel, a solution of ovalbumin and lysozyme was mixed at pH 5.3, then the pH of the solution was adjusted to 10.3, finally, the solution was stirred for 1 h, and heated at 80°C for 1.5 h, which resulted in the formation of a homogeneously dispersed nanogel. In this study, the author revealed that pH played a crucial role in the formation of homogeneously dispersed particles. For example, the isoelectric point (pI) of ovalbumin and lysozyme remain at acidic and basic pH, respectively, hence, under strong acidic or basic conditions, the resultant mixture was found to be transparent due to the formation of soluble complexes. NGs were found to be transparent even without the existence of complexes due to electrostatic repulsion between lysozyme and ovalbumin proteins. Mixing of both solutions at neutral pH induces precipitation due to strong electrostatic attraction. Morphology and particle size studied by TEM, revealed its spherical shape, however some aggregates also appeared which might occur during the evaporation of water content. The average diameter of particles was found to be 80 nm (dried particles), determined by TEM, while DLS revealed the real hydrodynamic diameter at 224 nm (Yu et al., 2006).

##### Self-Assembling Nanohydrogel From Soy Protein

Chen et al. fabricated and characterized stable nanogels of soy protein. As per the reported optimized procedure: soy protein with a dispersion strength of 1% w/v was heated at pH 5.9 at 95°C, without stirring for around half an hour. Further, the solution was immediately cooled to 4°C resulting in the formation of a nanogel. In the pH range of 2.6–3.0 and 6.0–7.0, the nanogel showed a low polydispersity index (∼0.1), and it was found to be stable from a 0–200 mM concentration of NaCl.

Formation of the stable nanogel was found to be dependent upon the denaturation of soy glycinin. The nanogel had a core-shell structure, in which the core constituted the β subunits bearing basic polypeptides (stabilized mainly by disulfide bonds as well as hydrophobic interaction) while shells constituted acidic polypeptides and α subunits. Overall, the fabricated nanogel was found to be stable against a wide range of pH and ionic strength change. The study proposed the good application potential of fabricated nanogels in the pharmaceutical and food industries as biomaterials (Chen et al., 2014).

#### Protein Nanohydrogels Prepared by Chemical Methods

##### Hydrogel Preparation by the Cold Gelation of Whey Proteins

The capability of whey proteins to undergo gelation under cold conditions were exploited for entrapment and delivery of diverse types of bioactive components and nutraceuticals. In a similar approach, Sadeghi and group took thermally treated whey protein isolate (WPI), calcium powder, and glucono-δ-lactone (GDL), and subsequently emulsified all ingredients with sorbitan monooleate in sunflower oil. Partial hydrolysis of GDL produced gluconic acid which induced gelation of whey protein by micro-emulsion under a cold condition, followed by centrifugation, which further enhanced precipitation. Particulate protein in the nanogel displayed new disulfide bonds in its structure. The study concluded that the nanogel matrix prepared by micro-emulsification can be explored for controlled release of bioactive compounds (Sadeghi et al., 2014).

##### Preparation of Soy β-conglycinin−dextran Nanogels via Self-Assembly Approach

Feng *et al.* fabricated a nanogel by conjugated soy β-conglycinin–dextran and performed its characterization and stability studies. To prepare the nanogel, first β-conglycinin was covalently conjugated with dextran via a Maillard dry-heating reaction which resulted in the formation of an amphiphilic graft copolymer. Further, this polymer was heated above the denaturation temperature at pH 4.8 to afford formation of the nanogel with a 90 nm size. The morphology studies illustrated the core-shell structures with spherical shape. Circular dichroism (CD) spectroscopy and surface hydrophobicity indicated distortion in the structure of β-conglycinins which exposed the hydrophobic groups at the protein surface and ultimately created hydrophobic compartments in the cores. The nanogel remained stable and intact in tested conditions against change in pH, temperature, lyophilization, and storage. Overall, the author concluded that the fabricated nanogel may be utilized for the delivery of hydrophobic bioactive compounds (Feng et al., 2015).

### Protein Nanohydrogel Reported for Specific Applications

**FIGURE 2:**
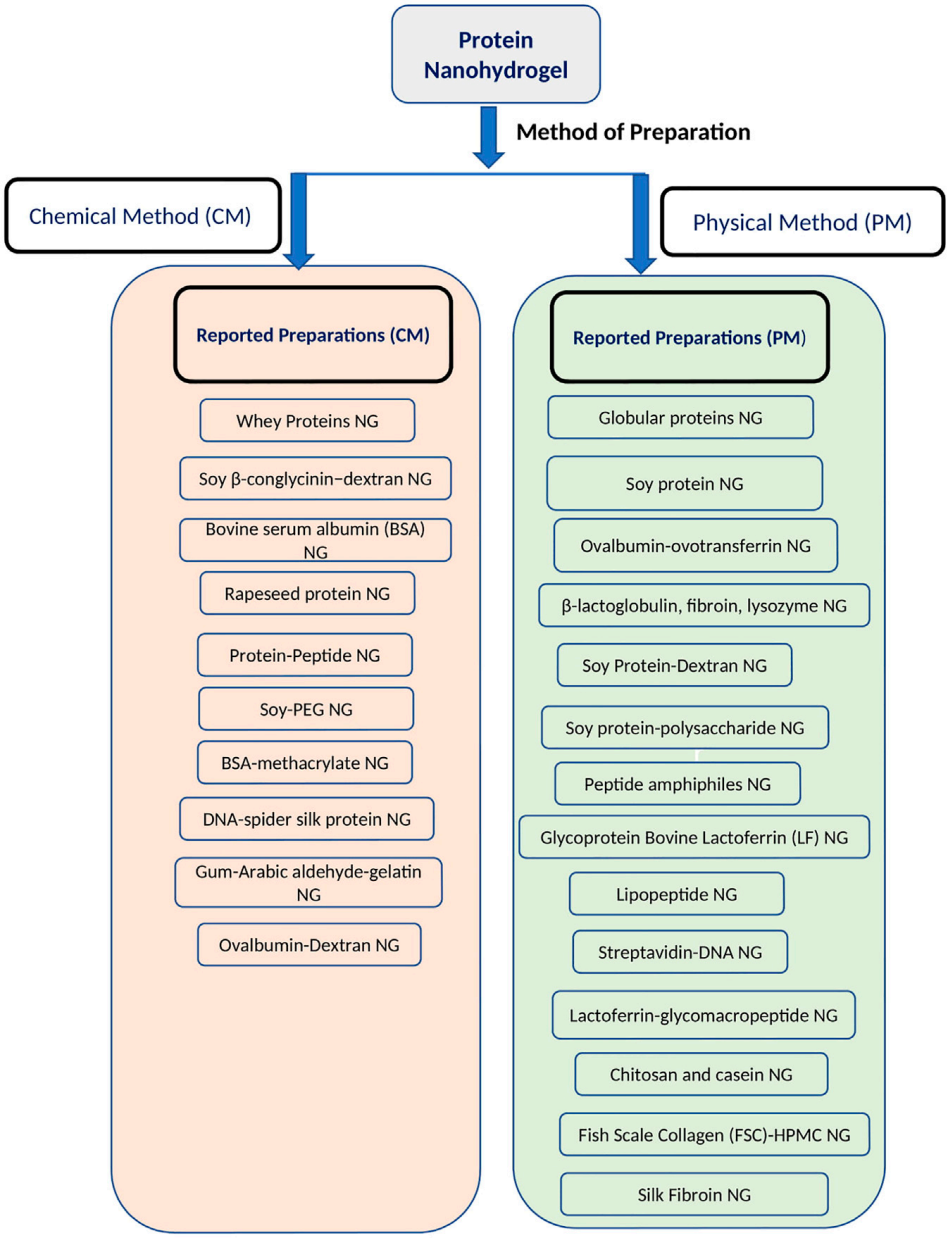
Reported protein nanohydrogels for bioactive delivery based upon methods of preparation.

Protein nanohydrogels generally comprise a network of nanofibers which create a reservoir for drugs and other bioactive compounds. Bioactive compounds either diffuse from this reservoir or are released upon the degradation of the nanohydrogels. The release profile of the bioactive compounds can be tactfully adjusted by changing the architecture of the hydrogel and microenvironment. By synchronizing the different factors like the nature of the constituent protein, its concentration, combination of other polymeric systems, pH, ionic concentration, or other environmental physicochemical stimuli, the release profile of bioactive compounds can be tuned (Bibian et al., 2015; Wang Q. et al., 2019). This section mainly emphasizes the preparations and applications of protein- or peptide-based nanohydrogels in bioactive delivery. Based upon the method of preparations, this section is also divided into two sub-sections (Figure 2).

#### Protein Nanohydrogels for Bioactive Delivery Prepared by Physical Methods

##### Nanogels of Ovalbumin-Ovotransferrin and Their Drug Loading Efficiency

Ovotransferrin (OT) and ovalbumin (OVA) are two major proteins present in the white portion of the hen egg. Hu et al. fabricated nanogels of ovalbumin and ovotransferrin using a solvent-free convenient approach. As per the optimized procedure, an aqueous solution of both proteins was prepared separately, then the OVA solution was added dropwise in the OT solution under the ice-cooled condition with an overall weight ratio of 1:1. The mixture was shaken gently and its pH was adjusted to around 7 to 7.5 using a 0.1 M solution of sodium hydroxide. Further, the mixture was heated at 80°C for half an hour, which resulted in a homogeneously dispersed nanogel. Characterization of the nanogel performed using DLC, TEM, and AFM revealed the spherical shape in a hydrated as well as in a dry state with a hydrodynamic diameter ranging from 100 to 220 nm. DLS studies provided information about the nanogel in its hydrated state, while dried state data were provided by the images of TEM.

The hydrodynamic diameter of the nanogel was found to be dependent upon the protein concentration and the method of preparation. The nanogel possessed a pH-dependent charge (positive below 5.5 and negative above 5.5) and displayed an amphoteric property. The fabricated nanogel exhibited stability in the pH range 2.0–4.0 and 7.0–11.0, however aggregates of dispersible nature appeared between pH 5–6. Loading efficacy of the nanogel was studied using benzoic acid as a model drug that remained in the range of 4.7–19% depending upon the pH, concentration of benzoic acid, and ionic concentration of NaCl.

In absence of salt, the nanogel showed maximum loading efficiency at pH 4, which might be possible due to the strong electrostatic interaction of benzoic acid with the nanogel at this pH. Further, the author revealed that benzoic acid did not bind with ovalbumin or ovotransferrin in the native states, but the nanogel of proteins could bind with the benzoic acid mainly with electrostatic and hydrophobic interactions (Hu et al., 2007).

Overall, the study reported the method of fabrication of a nanogel using OVA and OT without the use of solvents, demonstrated stability, and successfully loaded benzoic acid as a model drug. However, further investigations are required to test the stability upon long time storage, drug release pattern as well as drug release mechanism in *in vitro* and *in vivo* models. Furthermore, drug loading versatility of the nanogel should be investigated using other hydrophilic model drugs.

##### Protein Nanogels From Droplet Nanofluidic for Intracellular Delivery

Microscale hydrogels were explored for potential application in cell encapsulation, tissue engineering, and release of cargo molecules. They were found to be unsuitable for targeting intracellular delivery due to poor cellular uptake due to their size in micrometers. For intracellular delivery, Toprakcioglu et al. generated monodisperse nanosized w/o emulsions of three proteins: β-lactoglobulin, reconstituted silk fibroin, and lysozyme via a nanofluidics-based strategy with particle sizes ranging from 51 to 2,500 nm. In this study, the size of the particle was found to be inversely proportional to the flow rate ratio of the continuous phase (Q_cont_) and disperse phase (Q_dis_) in a nanofluidic device. The smallest particles size (up to 50 nm) was achieved by keeping the Q_dis_ constant (10 μL/h) and increasing Q_cont_ up to 60 μL/h.

Prepared nanosized particles incubated for self-assembly via the creation of a fibrillar network, followed by de-emulsification and re-emulsification resulted in the formation of the corresponding nanogels. Penetration studies performed using the nanogel on mammalian ovarian cancer cells revealed its good penetration ability across the membranes as well as delivery of intracellular cargo. The author concluded that biocompatible protein nanogels prepared by the facile and versatile route are highly suitable for storage and release of drugs to their intended destination (Toprakcioglu et al., 2020). The next phase of studies are required to establish the safety, metabolic stability, and pharmacokinetic profile of the nanogels.

##### Self-Assembling Soy Protein-Dextran Nanogel for Riboflavin Delivery

Jin et al. fabricated a self-assembled nanogel using modified soy protein and dextran. The nanogel was further characterized and studied for the encapsulation and delivery of riboflavin. To prepare the modified soy protein, it was denatured by heating at 60°C for half an hour, to this, dextran was added and mixed by ultrasonication for 70 min, which resulted in formation of the nanogel by a self-assembling process. Formation of the nanogel was confirmed by X-ray photoelectron spectroscopy (XPS), FT-IR, and zeta potential studies. The size of the nanogel was found in the range of 32–40 nm with spherical shape.

The size of prepared unloaded NGs varied with change in the pH of the system. The average size of NGs decreased when the pH reduced from 10 to 6, and it changed slightly between pH 6–5. Further dropping of pH from 5 to 2, resulted in an increase in size again due to increased electrostatic attraction, leading to large aggregates. Turbidity of NGs increased as pH deviated either below or above the isoelectric point of soy protein. At pH 6, NGs showed unimodal size distribution, which was selected as the optimal pH for further studies.

Stability of the nanogel remained intact after incorporation of riboflavin, and also upon changing environmental conditions up to a certain extent. Moreover, the size of the nanogel did not change significantly upon encapsulation of the riboflavin.

Treatment of the nanogel with a 250 mg/L solution of riboflavin exhibited around 65.9% encapsulation efficiency and release of riboflavin was found to be faster in simulated intestinal fluid (SIF) compared to simulated gastric fluid (SGF). Overall, the study demonstrated that nanogels prepared from modified soy protein-dextran showed nanoscale size, low size distribution, good encapsulation efficiency as well as loading capacity, and were able to deliver the riboflavin in its intact form (Jin et al., 2016). Drug loading studies in this report are limited to riboflavin which is quite hydrophilic in nature. Other hydrophobic drugs can be tested for loading and delivery which can explore the versatility of the delivery system. Moreover, drug release studies are performed to an *in vitro* level using simulated fluids but *in vivo* studies are lacking. Moreover, the *in vivo* profile may vary significantly from the *in vitro* results due to changes in environment particularly due to the presence of other food components and hydrolytic enzymes.

##### Soy Protein-Polysaccharide Nanogels for Folic Acid Delivery

The stability of folic acid is influenced by certain parameters like pH, temperature, and light (Gazzali et al., 2016). In a study, Ding and Yao reported nanogel preparation consisting of a soy protein/soy polysaccharide complex, and subsequently, its evaluation for the intestinal delivery of folic acid without degradation. Nanogels were fabricated via self-assembly of soy proteins with soy polysaccharides and folic acid under an acidic condition (pH 4.0) and subsequently by the denaturation of proteins via heat treatment. Polysaccharide-containing surfaces of the nanogels remained dispersible in the acidic environment while soy protein and folic acid being insoluble formed precipitates upon heating.

DLS studies confirmed the spherical shape of fabricated nanohydrogels while the hydrogel structure of NGs was confirmed by the swelling ratio, measured by the ratio of average volumes determined by DLS (VDLS) and atomic force microscopy (VAFM).

The soy protein and polysaccharide showed prominent electrostatic interactions in between the pH range 3.0–5.3. Upon storing the nanogels in NaCl solution (0.2 mol/L) at different pH values, the size increased from pH 4 to 5, while no significant change in size was observed in NGs stored at pH 2, 3, 6, 7, and 8. Upon rehydration, the lyophilized form of NGs regained their original size as in a freshly prepared nanogel. The fabricated nanogel not only protected folic acid from decomposition due to harsh conditions like acidic pH, heat, light, and oxygen but also allowed the release of undecomposed folic acid at neutral pH. The author stated that such a nanogel can be utilized for the delivery of folic acid while protecting it from acidic beverages and food (Ding and Yao, 2013). One limitation of this study was the lack of *in vivo* studies, moreover release studies were performed at the acidic and neutral pH conditions.

##### Peptide Amphiphile Nanofiber Gel for Cellular Delivery

The application of nanofiber gels comprising peptide amphiphiles has been investigated in the field of tissue engineering (Jonker et al., 2012). In one such study, Webber et al. constructed peptide amphiphiles conjugated with cell adhesion RGDS epitopes and evaluated this scaffold for the delivery of progenitor and stem cells derived from bone marrow.

In the *in vitro* studies, a system containing binary peptide amphiphiles with RGDS-containing molecules was found to be optimal for promoting cell adhesion, while a system containing only non-bioactive diluent showed 70% of the optimal growth. Further, adhesion was not increased upon adding RGDS in soluble form, which confirmed that promoted cell adhesion in the binary amphiphile system is specific to RGDS. In the *in vivo* studies, co-administration of a binary RGDS nanogel with luciferase-expressing cells via a subcutaneous route showed an around 3.2-fold increase in bioluminescent signal which confirmed the proliferative or anti-apoptotic property of the RGDS nanogel. *In vitro* and *in vivo* studies demonstrated the viable and proliferative nature of tested cells.

Further studies revealed the biodegradable and biocompatible nature of peptide amphiphiles without any significant tissue reaction. This study proposed the application potential of peptide amphiphiles as a biomaterial for cellular delivery in bone marrow-related disorders (Webber et al., 2010).

##### Lipopeptide-Based Nanohydrogel in Brain Drug Delivery

Certain lipopeptides consisting of lipid motifs connected with peptide chains have an ability to acts as bio-surfactant agents and undergo self-assembling in aqueous solution (Castelletto et al., 2017). Rambo and co-workers demonstrated pH and concentration-dependent self-assembly behavior of N-terminal lipidated heptapeptides. However, the parent peptide without the lipid part did not show self-assembly behavior. At neutral pH and high concentration, lipidated heptapeptide turns into hydrogel, while at different pH systems it shows different aggregated structures like fibrils, micelles, and nanosheets (Hutchinson et al., 2019).

In another study, Adak et al. developed a lipopeptide-based biocompatible hydrogel, consisting of hydrophilic amino acids, hydrophobic long chains, and microtubule-stabilizing peptides. Due to amphiphilic behavior, components undergo gel formation via a self-assembly process. The hydrogel showed efficacy as a neuroprotectant against anti-NGF-induced toxicity. In conclusion, the study proposed the good application potential of the hydrogel for delivery of hydrophobic drugs in the brain, particularly for patients of Alzheimer's disease and brain tumors (Adak et al., 2017 and Adak et al., 2019).

##### Release Behavior of Curcumin-Loaded Lactoferrin Nanohydrogels Into Food Simulants

Protein nanohydrogels are also being explored for the delivery of nutraceuticals. Araujo et al. (2020) prepared a nanohydrogel using the glycoprotein bovine lactoferrin (LF) and encapsulated curcumin as a lipophilic nutraceutical model. In this study, interaction of curcumin with the LF nanohydrogel along with its encapsulation efficiency and loading efficiency were evaluated. Furthermore, the release pattern of curcumin from the nanohydrogel were studied in the presence of a hydrophilic as well as hydrophobic food stimulant model with 10 and 50% ethanol, respectively and also in the presence of a gelatin matrix as a food model.

In this study, entrapment efficiency and loading capacity of the LF nanohydrogel were found to be 90 and 3%, respectively at a 80 μg/ml concentration of curcumin. The prepared LF nanohydrogel exhibited a spherical shape with size in range of 50–90 nm, and the shape was retained after loading of the curcumin. Spectroscopic analysis revealed that curcumin and LF bind via hydrophobic interactions with an average binding distance of 1.91 nm. The encapsulated drug system showed stability up to 14 and 35 days at 25 and 4°C, respectively. The system showed higher curcumin release in the presence of the lipophilic food stimulant (50% ethanol) compared to the hydrophilic stimulant (10% ethanol). Furthermore, the LF-curcumin system was found to be stable in the gelatin matrix which was taken as the food model.

Overall, the study revealed high encapsulation efficiency and loading capacity and proposed LF nanohydrogels as a promising vehicle for the controlled release of lipophilic nutraceuticals. Further, the author concluded that an LF-curcumin nanohydrogels system could provide fundamental information to deliver hydrophobic nutraceuticals in the gelatin matrix and its application can be extended for the delivery of the other similar nutraceuticals or functional foods in real food matrices (Araujo et el., 2020). But certain factors cannot be ignored when translating such a system for the *in vivo* delivery of similar nutraceuticals. For example, LF-curcumin nanohydrogels exhibited degradation with rise in temperature and were only found to be stable for 14 days at 25°C, while this study lacks stability evaluation at the higher temperature, so at a higher temperature such as body temperature (37°C), nanogel stability may be further hampered. Araujo et al. reported *in vitro* simulated curcumin release using a gelatin matrix as a food model but lacked *in vivo* studies, moreover, other factors are also not considered such as pH, presence of other food components, and digestive enzymes of the GIT, which can alter the stability as well as the release pattern of curcumin from LF-curcumin nanohydrogels (Knipe et al., 2015).

##### Streptavidin-DNA Nanohydrogels for Anticancer Drug Delivery and Cell Imaging

DNA has also been widely explored to develop biocompatible hydrogels for bioactive delivery and other biomedical applications. Apart from stability, the other main limitation associated with DNA-based hydrogels is their uncontrollable aggregation to form bulky-sized hydrogels, which ultimately limits its ability as an intracellular transport carrier.

**FIGURE 3:**
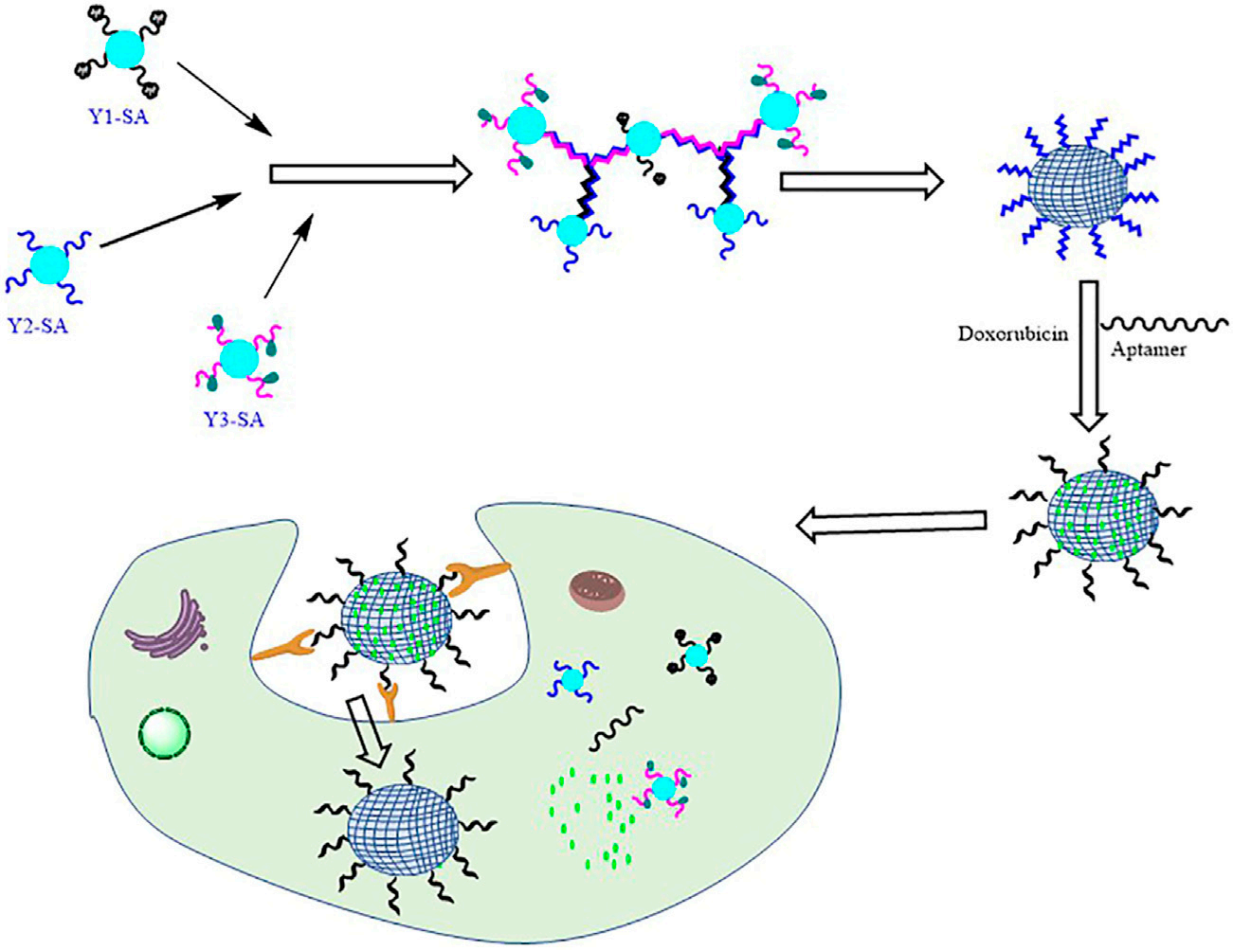
Streptavidin-DNA nanohydrogel for anticancer drug delivery (figure adapted with permission from Li et al., 2019, Copyright 2019, American Chemical Society).

In a recent study, Li et al. (2019) reported a strategy to develop multiple functional DNA-protein nanohydrogels of tunable size and explored its application in targeted anticancer therapy and cells imaging (Figure 3). To fabricate the nanohydrogel, three different types of streptavidin (SA)-based DNA tetrads were prepared. The three SA-DNA tetrads, abbreviated as Y1-SA, Y2-SA, and Y3-SA, consisted of streptavidin and four DNA probes. Y1 consisted of DNA with a special binding affinity to the ATP while Y2 and Y3 consisted of a fluorescence donor (Cy5) and acceptor DNA probes, respectively. The loose and flexible structures of SA-DNA tetrads lead to transformation into a hybrid streptavidin DNA hydrogel (SDA). In this study, the size of the hydrogel was made in nanoscale by controlling the initial concentration of the DNA tetrad.

The size and morphology of the SDH were investigated by atomic force microscopy (AFM) and dynamic light scattering (DLS). In the DLS studies, the diameter of DNA-SA was found to be 10.1 nm which increased up to ten times (103.2 nm) after the self-assembly of DNA tetrads, while the image of AFM revealed their monodispersed spherical structure in the nanoscale range. SDHs were loaded with anticancer drug doxorubicin and tested for tumor-specific delivery, these were further modified with a unique aptamer known as MUC1, which have special recognition and binding affinity to the MUC1 glycoprotein which are generally overexpressed on the surface of many cancer cells.

Functionalized SDHs with doxorubicin and MUC1 showed high affinity toward the targeted cancer cells and entered inside the cell via receptor-mediated endocytosis. In response to the high ATP level inside the cancer cells relative to healthy cells, DSP disassembled and released the loaded doxorubicin preferentially in cancer cells, in parallel it also displayed strong fluorescence and ultimately induced apoptosis. Functionalized SDHs were found to be biocompatible, had the ability of tunable size, and functional modification. Overall, the study reported a novel ATP-activatable DNA hydrogel for targeted anticancer therapy and imaging. The authors concluded that such a programmable and stimuli-responsive nanohydrogel can be explored further as a cancer therapeutic (Li et al., 2019), however, this study was confined to the *in vitro* level and its execution to an *in vivo* level necessitates additional studies, such as the suitable route of administration, immunogenicity, general toxicity, metabolic stability, and suitable pharmacokinetic profile.

##### Chitosan-Coated Lactoferrin-Glycomacropeptide Nanohydrogel for Drug Delivery

Bourbon et al. prepared nanohydrogels of lactoferrin-glycomacropeptide (Lf-GMP) and subsequently coated them with chitosan. The degradation of the chitosan-coated nanoparticles as well as bio-accessibility were tested using a dynamic *in vitro* digestion model. Chitosan coating reduced the degradation of Lf and GMP by 10 and 18%, respectively compared to the non-coated batch. Further, protein bio-accessibility studies revealed that 28 and 40% of Lf and GMP remained stable until absorption into the chitosan-coated nanohydrogel formulation. In order to evaluate the delivery ability of the chitosan-coated Lf-GMP, hydrophobic molecule curcumin and hydrophilic caffeine were encapsulated and the bio-accessibility of both drugs was tested in an *in vitro* digestion models in comparison to its free forms. The result of the study revealed that bio-accessibility of curcumin and caffeine was enhanced to 72 and 63% in comparison with their free state (66 and 59%, respectively). Moreover, the study revealed that the free form of curcumin lost around 68% of its anti-oxidant property in the digestion model while the chitosan-coated Lf-GMP nanohydrogel loss only 30% of its activity.

The study proposed that chitosan not only reduced the enzymatic degradation of the nanohydrogel in the intestine but also enhanced its stability against gastric digestion by forming a hydrogel in the presence of gastric hydrochloride acid. This study concluded that chitosan coating over protein-based nanohydrogels can improve the stability and bio-accessibility of proteins as well as for encapsulated drugs preferentially of hydrophobic nature (Bourbon et al., 2018).

Two general limitations of this study are relevant to the preparation methodology and drug loading efficiency of the nanohydrogel. Although the preparation of nanogels seems simple, it involves heating at 80°C post loading of the drugs, therefore, this method may not be desirable for loading other thermosensitive drugs. The study lacked a discussion on drug loading capacity of the nanogel, although the encapsulation efficiency was reported around 95 and 90% for curcumin and caffeine, but it was at quite a low concentration of the respective drugs (82 and 30 μg/ml, respectively).

##### Functionalized Chitosan and Casein Nanohydrogels for Drug Delivery

Du et al. reported self-assembled nanohydrogels (NGs) for the delivery of EWDP (egg white-derived peptide) and curcumin as a model of hydrophilic and hydrophobic drugs, respectively. Nanohydrogels NAC-CS-CA NGs and CYS-CS-CA NGs were prepared using the N-acetyl-l-cysteine (NAC) and l-cysteine (CYS)-functionalized chitosan (CS) with treated casein protein (CA). The size of the NGs was found to be dependent upon the pH and both NGs showed better stability in the pH range of 2–5. Dynamic light scattering studies revealed the smallest size of NGs in nanomolar ranges, 89 and 86 nm for NAC-CS-CA NGs and CYS-CS-CA NGs, respectively at the 4 pH value. Zeta potential of both NGs were found to be >30 mV at the pH range of 2–5 which indicated the presence of strong electrostatic repulsion, and could be accounted for by the stability of the system in this pH range. NGs were found to be stable for 21 days at room temperature. The entrapment efficiency (EE) for both model drugs varied significantly with change in pH value. EE of ESDP was found in the range of 39–67% and 45–58%, and for curcumin it was 51–63% and 48–63%, in the NAC-CS-CA NGs and CYS-CS-CA NGs, respectively. Entrapment efficiency of EWPD and curcumin was found to be optimum around pH values of 3 and 4, respectively in both NGs. FT-IR spectroscopy revealed hydrogen bonding as a common entrapment mechanism of both drugs. Additionally, strong electrostatic and hydrophobic interactions were found specific to EWDP and curcumin, respectively. Sustained release of EWDP and curcumin was reported from the NGs for 56 and 80 h, respectively. Overall, the scientist concluded from the study that the NGs based upon NAC/CYS-CS-CA may act as potential nanocarrier systems to deliver bioactive compounds of diverse polarity (Du et al., 2019).

Talking about the limitations of this study, simulated fluids without enzymes were used to study the release profile of the EWDP and curcumin, which does not exactly mimic the GIT environment *in vivo*. Secondly, encapsulation efficiency of both model drugs were not found be very promising and varied from 39 to 67%. Preparation of the NAC/Cys conjugate involved use of coupling agents EDC and NHS, hence, unseparated traces of such reagents may contaminate the nanogel system and consequently hamper the biological application.

##### Curcumin-Loaded Fish Scale Collagen (FSC)-HPMC Nanogel for Wound Healing

Pathan et al. prepared a curcumin-loaded fish scale collagen (FSC)-hydroxypropyl methylcellulose (HPMC K100) nanogel (CNG) and evaluated it for wound healing properties. In this study, curcumin in the nano-emulsion form was loaded into the FSC-HPMC. The nanogel was characterized and tested for stability, skin irritation, and efficacy studies. The droplet size and PDI of the optimized formulation was found to be 123 nm and 0.155, respectively, determined by photon correlation spectroscopy (Delsa Nano C).

CNG showed prolonged release of curcumin in the permeation studies performed on human cadaver skin and efficacy studies in Wistar rats demonstrated a higher contraction value of the wound as compared to the control formulation. The tested nanogel did not show significant skin irritation with a score of less than 2 compared to the control group. Overall, the study concluded the long-time stability, good permeation as well as wound healing ability of curcumin-loaded FSC-HPMC. FSC has abundant natural sources, however its isolation and purification are tedious processes which require treatment with several reagents, moreover its vulnerability toward the microbial growth cannot be ruled out (Pathan et al., 2019).

##### Silk Fibroin Nanohydrogel in Delivery of Bioactive Insulin

Maity et al. designed and prepared an injectable form of a silk fibroin hydrogel (iSFH) in order to provide the sustained delivery of insulin in a diabetic condition. The silk fibroin protein hydrogel prepared in combination with ethylene glycol and triethylene glycol was found to be porous in nature which encapsulated insulin in its active form.

Administration of iSFH-encapsulated insulin via subcutaneous route to type 1 diabetic Wistar rats maintained the glucose level for up to 96 h and confirmed the controlled and slow release of insulin.

The gelation time of the SFH was determined by measuring the change in the OD at 550 nm while circular dichroism (CD) was used to analyze the change in the secondary structure of the protein in SF and iSFH.

While exploring the gelation mechanism, the author proposed that added viscous glycols restrict the mobility of the SF backbone and induces transformational changes in the secondary structure of SF from a random coil state to a β-sheet structure, which results in the gelation of protein. Weak van der Waals interactions were confirmed between the SF protein and the added glycols in the iSFH matrix (Wang et al., 2015). Lower swelling ratio allowed for slow release of insulin without burst effect, so that insulin-iSFH injection did not cause hypoglycemia. The hemolysis studies performed using iSFH on human RBC revealed no detectable cell lysis, while in biocompatibility studies performed on L929 fibroblast cells, iSFH showed around 93% excellent cell viability compared to the control group.

Overall, excellent biocompatibility, good mechanical strength, and ability of sustained insulin delivery indicated the good application potential of iSFH-encapsulated insulin for diabetic patients, which can be further preceded in advanced studies (Maity et al., 2020).

#### Protein Nanohydrogels Prepared by Chemical Methods and Role in Bioactive Delivery

##### Self-Assembling BSA Protein Nanogels for Cancer Immunotherapy

Protein-based nanogels are investigated as versatile vehicles in vaccine development. Purwada et al. prepared a vaccine involving a self-assembly protein nanogel and utilized it for delivery of a soluble antigen to produce robust immune cell response. Singh et al. synthesized a poly (hydroxyethyl methacrylate) (pHEMA)-pyridine polymeric system using dimethylaminopyridine (DMAP) (Singh et al., 2014) which upon self-assembling with bovine serum albumin (BSA) at pH 7.4 produced a nanogel in the size range of 145–160 nm. Ovalbumin was incorporated as a model vaccine antigen in the prepared nanogel.

Loading capacity of the nanogel increased upon increasing the concentration of BSA, however at a 60 μg ml^−1^ concentration, a nanogel was obtained with maximum particle count and fair loading ability. The uptake of the nanogel in the DC population was studied by flow cytometry measuring the fluorescence.

Nanogel formulation did not show significant toxicity in dendritic cells (DCs) at tested concentration (33.75–150 μg/ml). The nanogel-based vaccine was successfully processed by the DCs and showed good antigen presentation. The study proposed that a similar approach could be utilized for the delivery of other soluble antigens or biomolecules with poor tissue retention as immunomodulators. The study concluded that self-assembled nanogels are versatile systems in which biomolecules or therapeutics can be incorporated without the use of other organic solvents and emulsifying agents (Purwada et al., 2016).

##### Self-Assembling Rapeseed Protein Nanohydrogel for Delivery of Curcumin

Wang et al. fabricated a self-assembled nanogel from the proteins of rapeseed as a carrier system for the delivery of hydrophobic drugs. In this study, acylated rapeseed protein isolate (ARPI) was prepared from rapeseed protein isolate by acylation reactions. The solution of ARPI was heated at 90°C, i.e., above denaturing temperature (80°C) for 30 min and cooled immediately to obtain the ARPI nanogel. The effect of other factors like pH, temperature, heating, and concentration of ARPI were investigated upon the physicochemical properties of nanogels. The AFM image and DLC studies of optimized nanogels revealed the spherical shape with a 170 nm hydro diameter, respectively. Secondary and tertiary structure of the protein in the ARPI nanogel evaluated by circular dichroism showed a significant difference from the native rapeseed protein isolates as well as ARPI without the heat treatment. Moreover, the ARPI nanogel exhibited increased hydrophobicity with reduced content of the free sulfhydryl group. Overall, covalent disulfide bond formation created a hydrophobic core in ARPI gel, while newly attached succinyl groups remained exposed toward the water in the shell. The cross-linked structure of ARPI nanogels was found to be resistant against a wide range of ionic concentration, pH, lyophilization process as well as dilution.

The encapsulation efficiency of ARPI nanogels was investigated using curcumin as the hydrophobic protype which was found to be 95%, and no significant change was observed in the size of nanogels upon loading the curcumin. ARPI nanogels significantly increased the solubility of poorly soluble curcumin by more than 100 times, and also enhanced its anticancer activity against several cancer cell lines (Wang Z. et al., 2019).

At the preliminary level, the study explored the potential of ARPI nanogels as a drug delivery system, a few aspects which need further investigation are stability studies in storage conditions, possible route of administration, and *in vivo* studies to establish the pharmacokinetic and pharmacodynamic profile of the nanogels. In addition to this, possible toxicities of the acylating reagent, i.e., butanedioic anhydride or its degrading products which may remain in trace amounts also need to be assessed before *in vivo* testing.

##### Protein-Peptide Hydrogels in Cell Adhesion

Shroff et al. developed a peptide amphiphile mimetic to fibronectin with sequences GRGDSP and PHSRN. Among this, GRGDSP functions as a cell-binding domain, while PHSRN was incorporated to increase the specificity and activity of the former. Using the fibronectin mimetic, they synthesized peptide PR_g [GGGSSPHSRN(SG)5RGDSP] bearing the GGGSS sequence as a spacer. PR_g was linked with the C16 hydrocarbon tail to get peptide amphiphiles, which in deionized water produced hydrogels by self-aggregation. Prepared hydrogels showed excellent cell adhesion as well as proliferation when tested on endothelial cells of the human umbilical vein. Moreover, cells on peptide amphiphile gels also promote fibronectin secretion, an extracellular natural adhering protein. Based upon the key findings of this study, it can be concluded that hydrogels derived from peptide amphiphile can be explored as ECM mimetic scaffolds having a long term cell adhesion property, and its similar application can be explored in tissue engineering (Shroff et al., 2010).

The fact that protein or polypeptide hydrogels can mimic an extracellular matrix (ECM) was investigated in tissue engineering and cell culture (Francis et al., 2016). Xu and co-workers developed an injectable hydrogel using poly (l-glutamic acid)-based and arginylglycylaspartic acid (RGD) (Xu et al., 2017). The study revealed that RGD incorporation to the polypeptide network enhanced cell proliferation and cell adhesion.

##### Biomimetic Soy-PEG Nanohydrogel for Wound Dressing

Snyders et al. prepared a biomimetic hydrogel using soy protein and poly (ethylene glycol) for moist wound dressing applications. Fabrication of the hydrogel was achieved by the reaction of activated PEG and the amino groups of the protein, to form stable urethane linkage which was subsequently washed with phosphate buffer for removal of unconsumed reactants. In order to study the structural network, mechanical properties, and protein absorption, the hydrogel was evaluated for tensile and unconfined compression measurements. Elastic moduli of the hydrogel were found to be dependent upon the composition of the hydrogel which ranged from 1 to 17 kPa. The elasticity of the material increased upon increasing the concentration of soy protein and a solution of 12% soy protein produced the strongest hydrogel upon polymerization. The author proposed a 3D model network of the hydrogel to explore drug release properties (Snyders et al.,2007).

##### Protein Nanogels in Conjugation With Antibodies

Matsumoto *et al.* reported a methodology for the preparation of protein-based nanohydrogels from bovine serum albumin (BSA) by the covalent conjugation. Polymeric nanogel precursors were synthesized by the copolymerization of pyridyl disulfide methacrylate (PDSMA) and poly (ethylene glycol) methyl ether methacrylate (PEGMA). Further, the reaction of dithiothreitol with p(PEGMA-*co*-PDSMA) afforded nanogels.

DiI dye as a model hydrophobic drug was loaded within the interior of the nanogel by a non-covalent interaction, subsequently reducing crosslinking agent DTT was added in order to entrap the dye. In the next phase, N-succinimidyl-S-acetylthiopropionate (SATP)-activated BSA was conjugated via formation of a new disulfide bond. The hydrodynamic diameter NG-DiI–BSA bioconjugates sample was found to be 25 nm.

Conjugation was confirmed by different techniques like dynamic light scattering, agarose, polyacrylamide gel electrophoresis, and protein liquid chromatography. Under the reducing conditions, the conjugated protein was released due to breaking of the disulfide bond leading to the release of free dyes. The study demonstrated that based upon a similar concept, other proteins can be conjugated to nanogels loaded with hydrophobic molecules. Such a polymeric nanocarrier can be used as a tool for dual purposes, i.e., diagnostic and therapeutic purposes (Matsumoto et al., 2013).

##### DNA-Spider Silk Nanohydrogels for Delivery of Thrombin

**FIGURE 4:**
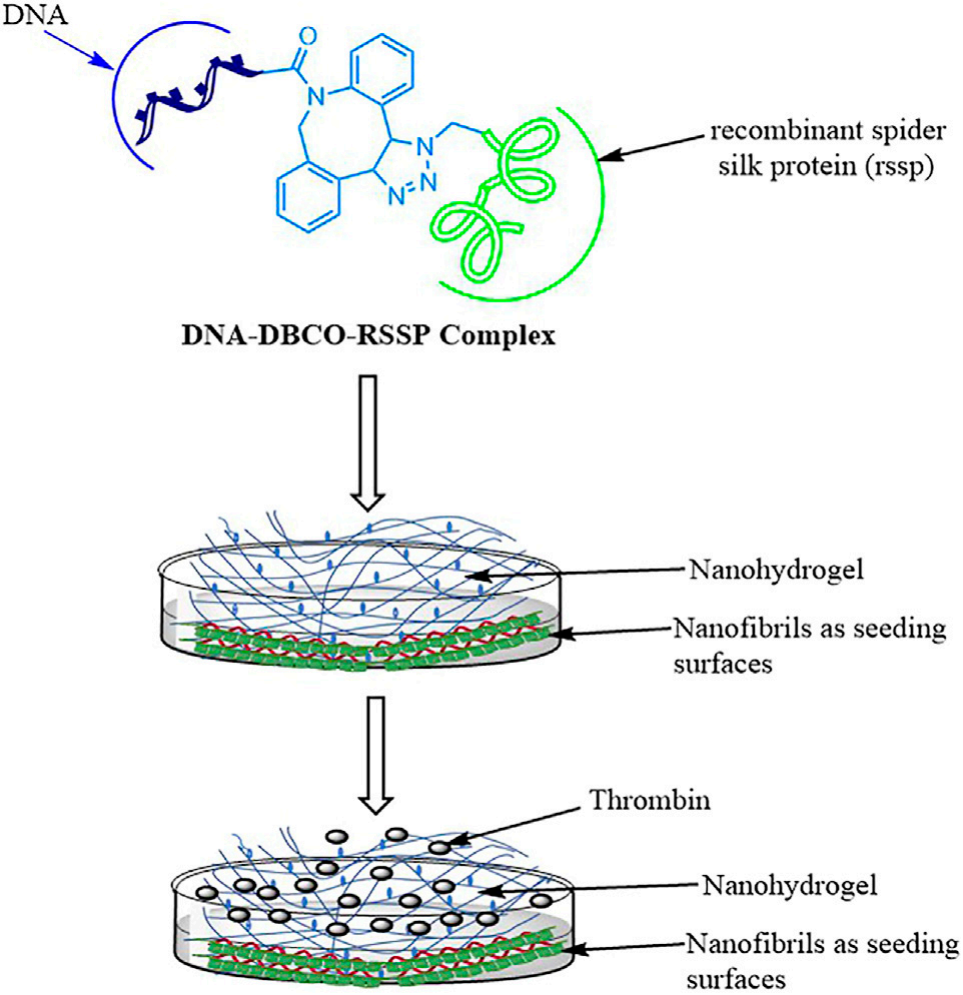
Preparation of DNA-spider silk protein-based nanohydrogels for delivery of thrombin (figure is modified and redrawn from the original work of Humenik et al., 2020).

A protein-based nanohydrogels matrix is also explored for encapsulation and release of sensitive enzymes. Humenik et al. prepared immobilized nanohydrogels using the recombinant spider silk protein (rssp), and prepared a nanohydrogel depot which was tested for the release of active thrombin. In this study, specific anti-thrombin DNA fragments were coupled with 5′-dibenzocyclooctyne, which provided a 5′-dibenzocyclooctyne-modified DNA complex (DNA-DBCO). The complex was then subsequently coupled with the azide-linked spider silk protein (N3-rssp), which afforded the hybrid complex (DNA-DBCO-RSSP). Subsequently, the spider silk protein (RSSP) self-assembled while the linked DNA folded into aptamers (apt), overall providing aptamer-functionalized nanofibrils (Figure 4). The surface coated with the above nanofibrils was utilized as a seeding surface for self-assembly of more-free RSSP which resulted in a confined nanohydrogel. Next, thrombin was further entrapped as a model enzyme inside the nanohydrogel which provided conditions mimicking blood plasma. The chosen aptamers in the study bound at the specific exo-sites of thrombin which enabled selective embedding of the thrombin into the nanohydrogels. In the next phase, addition of the complementary DNA sequences led to changes in the secondary structure of the incorporated aptamer, with subsequent release of the active thrombin for catalytic activity (Figure 4). The author proposed that a similar concept could be explored for the preparation of nanohydrogel depots which could be utilized for the storage and release of the active form of the sensitive enzyme on a on-demand basis (Humenik et al., 2020; Keefe et al., 2010).

##### Gum-Arabic Aldehyde-Gelatin Nanohydrogel for Anticancer Activity

Sarika et al. prepared and characterized curcumin-loaded nanohydrogels of gum-Arabic aldehyde (GA Ald)-gelatin (Gel) and evaluated their *in vitro* anticancer activity.

Gum Arabic is a natural polysaccharide which mainly contains arabinogalactan as the main monomeric constituent. The vicinal diols of arabinogalactan in GA can be oxidized to aldehyde groups which can form the imine bond with the free amino group. In the proposed study, it forms an imine bond with free amino groups of gelatin resulting in the formation of the nanohydrogel (Sarika and James, 2015). GA Ald-Gel nanogels were prepared by the mixing of the mini-emulsions of gelatin and GA Ald separately by the ultrasonication method and subsequent loading of curcumin by dissolving it in acetone (2 mg/ml). Curcumin-loaded hydrogels were characterized to determine the physicochemical properties by techniques like scanning electron microscopy (SEM), dynamic light scattering (DLS), and NMR. Characterization studies revealed negative zeta potential (−27 mV) with a hydrodynamic diameter of 452 ± 8 nm. The encapsulation efficiency of nanogels was found to be 65 ± 3% and exhibited controlled release, which was found more in acidic pH compared to neutral pH. *In vitro* cytotoxicity studies in the MCF-7 cells showed the cytotoxic effect of the nanogel, while confocal laser scanning microscopy (CLSM) studies confirmed its intracellular uptake. Overall, the study proposed the suitability of curcumin-loaded GA Ald-Gel for cancer therapy.

In the hemocompatibility assay, curcumin-loaded nanogels showed concentration-dependent hemolysis, but it was within a safe limit (>5%) as per the ISO/TR 7406 guidelines for biomaterial. Overall, the study proposed GA Ald-Gel as promising carriers for curcumin, which can be further explored for anticancer drug delivery (Sarika and Nirmala, 2016). However, use of strong oxidizing agents and reactive intermediates like aldehyde generated in the preparation process necessitate an optimized purification procedure to avoid possible associated toxicity.

##### Ovalbumin-Dextran Nanogel in Delivery of Curcumin

Feng et al. prepared conjugates of ovalbumin-dextran via chemical reaction and converted them into nanogels via the heat-induced gelation process. Characterization of nanogels revealed their spherical shape and confirmed the covalent bonding. Curcumin was loaded as a model molecule through a pH-driven method and its bioavailability was evaluated using an *in vitro* gastrointestinal tract model. Dextran of different molecular weights ranging from 10, 40, 70, and 150 kDa was used for conjugation with ovalbumin, and the study revealed that conjugation efficiency decreased with the increase in the molecular weight. The study proposed that the lower accessibility of reducing sugar upon increasing the molecular weight of dextran may be the possible reason for the decrease in the conjugation efficiency. The EE and LC of curcumin in the nanogel was found to be 82.15 and 6.92%, respectively. Incorporation of curcumin in the nanogel caused no significant change in the morphology and particle size. Curcumin transformation was found to be more significant as compared to the nanoparticle of ovalbumin, which indicated the better protection of curcumin from the degrading enzyme in the nanogel form. Curcumin nanogels and ovalbumin nanoparticles displayed almost same bio-accessibility. Overall, the finding of the study revealed that nanogel formulation has the potential to improve the bioavailability of curcumin in the simulated GIT models. Further study is required to evaluate the delivery ability of the system in an *in vivo* model (Feng et al., 2016).

### Advantages of Protein Nanohydrogels

Applications of natural protein-based hydrogels are being explored in different sectors due to certain unique properties like biocompatibility, good nutritional value, excellent functional properties, biodegradability, amphiphilic behavior, and fewer toxicities as compared to synthetic polymers (Snyders et al., 2007; Panahi and Baghban-Salehi, 2019). Protein hydrogels can protect incorporated cells, peptides, biomolecules, and drugs from the harsh environment. Nanogels loaded with active compounds can be injected into the body which can change its thixotropic property at body temperature and physiological pH (Mondal et al., 2020). Properties and sizes of nanohydrogels can be customized to avoid clearance by phagocytic cells and to reach the target by active as well as passive routes. Due to their nano size, these can be easily transported within the systemic circulation and reach deeper tissues by penetration or another mechanism. Protein nanohydrogels generally possess amphiphilic properties which can incorporate hydrophobic as well as hydrophilic drugs, including charged species (Abaeea et al., 2017). Proteins or peptides can easily undergo self-assembly in the absence of covalent binding agents, solvents, or other external reagents because various types of non-bonding interactions like ionic, hydrophobic, van der Waals, and pi-pi interactions, etc. can favor the self-assembling process (Yang et al., 2016).

### Disadvantages of Nanohydrogels

Despite several advantages of protein nanogels, there are certain drawbacks which affect their application potential. For example, during preparation, sometimes a small amount of the monomers or surfactants remain unconsumed in the nanogel, which may show toxic effects in the body. Removal of such surfactants, unreacted monomers, and solvents require expensive technology, which enhances the cost of nanogel preparation (Vinogradov, 2010). Usually, all desired properties are not attained uniformly or remain confined to a limited portion of formulation, which generally creates problems in the manufacturing of large batches. Protein hydrogels which are stabilized by non-covalent weak interactions are in general mechanically weak and show stability issues. Properties of nanohydrogels depend upon the folding and unfolding of the incorporated protein components, which itself is affected by certain environmental factors and ultimately may affect the steady drug release pattern (Schloss et al., 2016). In some cases, a prominent interaction between the nanogel polymeric system and the drug reduces the hydrophilicity of the formulation, which can irreversibly entrap the drug (Kabanov and Alakhov, 2002).

Other challenges associated with protein nanohydrogels are their susceptibility toward enzymatic digestion and degradation by gastric HCl especially when given by the oral route, the susceptibility mostly gets reduced by the coating of other polymers like chitosan (Bourbon et al., 2018). Hydrogels possess high osmotic pressure in the swollen state, which reduces their adhesiveness, particularly, toward biomolecules (Sudre et al., 2012). Upon loss of water content, a nanohydrogel may lose its re-swelling ability to attain the original volume, which is mainly attributed to the formation of new interactions and loss of water (Panahi and Baghban-Salehi, 2019).

### Toxicological Aspects of Protein Nanohydrogels

A nanogel possesses a large surface/mass ratio that may enhance the reactive capacity or also alter degradation rate. Sometimes, a nanogel carrier system interacts with cells which may abnormally damage or kill the cells. However, later effects of nanogels are being utilized in the delivery of anticancer agents (Ferrer et al., 2012; Ferrer et al., 2014; Coll Ferrer et al., 2014).

#### Toxicological Aspects of Protein NGs Prepared by Chemical Methods

Major concerns of chemical cross-linked protein NGs are toxicities associated with biomaterials, unreacted monomer units, coupling agents, solvents, side products, or other reagents. Therefore, in order to prepare safe protein NG-based delivery vehicles, one should also consider toxicity and metabolism of polymer materials, the nature of coupling reagents, solvents, other additives, and the possibility of side products generated during the chemical reaction, so that the prepared formulation has least deleterious effects on the body (Soni et al., 2016).

Synthetic NGs are frequently prepared by crosslinking of amine groups with aldehyde or carboxylic acid, therefore they require aldehydes or other chemical-based cross-linkers or oxidizing agents, which are generally toxic in nature (Panahi and Baghban-Salehi, 2019).

#### Toxicological Aspects of Protein NGs Prepared by Physical Methods

Nanogels consisting of stable proteins such as lysozymes and carbohydrates like dextran are considered to be free from prominent side effects. A natural protein like collagen is widely used in tissue engineering, however collagen and its degradation products are reported to have thrombogenic potential, which can trigger the adhesion of platelets via activating the coagulation cascade. Protein like elastin in pure form is considered biocompatible, however its purification process is tedious, therefore, impurities either remaining after purification or entering during the purification process may lead to undesirable immune responses (Catoira et al., 2019).

Another limitation of elastin is vascular calcification which mainly occurs due to its binding affinity with calcium resulting in the stiffness of blood vessels (Pai et al., 2011). Protein NGs from natural origin may transfer certain pathogens including viruses (Singhal et al., 2016). NGs comprising natural proteins grafted with synthetic polymers are emerging as hybrid NGs with good biocompatibility, stability, and other customizable features (Purwada et al., 2016; Panahi and Baghban-Salehi, 2019). The safety profile of some synthetic polymers like polyglycolic acid (PLGA), polylactic acid (PLA), and acrylates like methacrylate are already established which when hydrolyzed form non-toxic oligomers or monomers. So, hybrid NGs of such polymers with proteins can be explored for potential application in drug delivery and other biomedical applications (Langer and Tirrell, 2004; Calzoni et al., 2019).

### Stability Aspects of Nanogels

The versatile nature of the nanogel allows for the incorporation of a wide range of molecules, like organic drugs, active phytoconstituents, inorganic nanoparticles, and large biomolecules like proteins and DNA while retaining their gel-like behavior (Soni et al., 2016; Oh et al., 2009; Beija et al., 2011). Nanogels consisting of inorganic nanomaterials are generally used *in vivo* for diagnostic and imaging purposes, but these are associated with certain stability issues, like the short life of the colloidal system, poor solubility, and fast elimination by phagocytes. Nanogels based upon polymeric systems including peptides and proteins are more versatile, which can be made stable toward enzymes with long circulation half-lives and can be exploited as a good platform for the delivery of therapeutic molecules (Beija et al., 2011; Siegwart et al., 2012). Many multifunctional nanogels are fabricated for a site-specific response, the stability depends upon the specific stimuli, which are generally associated with certain pathological conditions and local environmental factors like temperature, pH, redox balance, and level of certain biomarkers or mediators (Morimoto et al., 2008; Pan et al., 2012; Cheng Y. et al., 2013; Xiong et al., 2012). Protein nanogels possessing disulfide bonds are highly sensitive toward the reductive difference in the intracellular and extracellular environment, such nanogels may remain stable in the blood only for a while, it further gets degraded and releases the drug inside the cell (Oh et al., 2007; Li et al., 2013).

Huppertz et al. performed the covalent bonded crosslinking of casein micelles using the enzyme transglutaminase to prepare the nanogel evaluating its stabilities under acidic conditions, heat, and different concentrations of MCP. Casein nanogel particles were found to be less stable under acidic environments, while it showed good stability against heat compared to native casein micelles. Further, stability toward acid-induced and heat-induced coagulation enhanced with decreasing the content of MCP (Huppertz and de Kruifa, 2008).

### Preclinical Studies and Clinical Potential of Nanogels

Nanogels have been reported as novel drug delivery vehicles in several diseases and pathological conditions including cancer. In such a study, Dorwal et al. injected the recombinant murine interleukin-12 (IL - 12) incorporated in the nanogel of cholesteryl pullulan in mice suffering from subcutaneous fibrosarcoma via the subcutaneous route. Treated mice showed elevated levels of IL-12 for a prolonged time and reduced the growth of tumors (Shimizu et al., 2008). Nanogels based upon cholesteryl pullulan showed good potential in the delivery of peptides in the clinical trials. In such a study, the HER-2 vaccine incorporated in the cholesteryl pullulan-based nanogel was administered to nine patients with a dose of 300 μg twice in a week via a subcutaneous route. All the patients showed good tolerance against vaccines with mild sensitivity at the site of injection. Moreover, all patients exhibited good CD4^+^ and CD8^+^ T cell responses, which demonstrated the good therapeutic potential of the vaccine (Dorwal, 2012). In another study, Lee et al. reported clinical trials on delivery of Aβ oligomers incorporated in cholesteryl pullulan nanogels taken by patients with Alzheimer’s disease. Studies demonstrated the better penetration capacity of Aβ oligomers with reduced toxicity to the nervous system cells (Boridy et al., 2009). *In vivo* application of antibiotics conjugated with nanogels have been tested in phase 1 clinical trials (Vinogradov, et al., 2004). Nukolova et al. (2011) used PEO-b-PMA diblock copolymers to prepare the nanogels. Folic acid in its activated state was conjugated with the nanogel via the terminal amino group. Nanogels were further loaded with anticancer drugs cisplatin or doxorubicin and administered to mice already fed with a folate-deficient diet. In the study, the treated mice group showed the prominent antitumor effect of cisplatin and also displayed less toxicity compared to the free drug. In another study, a silver nanoparticle nanogel of poly (4-vinylphenylboronic acid-co-2-(dimethylamino) ethyl acrylate) incorporated with optical sensitive insulin was designed for the management of diabetes, which opens a new era in the field of clinical trial (Wu et al., 2010).

Diverse types of hydrogel-based formulations are available in the market for clinical use, such as contact lenses, wound dressings, drug delivery, tissue engineering, and hygiene products but the majority of these involve the use of other synthetic polymers and most of them are polysaccharide-based hydrogels (Calo and Khutoryanskiy, 2015; Cascone and Lamberti, 2019; Gupta et al., 2019). Extensive literature analysis revealed that protein-based hydrogels are relatively less explored, particularly, protein nanohydrogels for bioactive delivery are reported only in limited studies, which are mainly confined to *in vitro* studies and only a few are conducted on animals (Dalwadi and Patel, 2015; Wang Q. et al., 2019; Panahi and Baghban-Salehi, 2019). However, conventional protein hydrogel-based formulations containing mainly gelatin and collagen are already approved for clinical use. These are mainly approved for some allied indications like correction of soft tissue, spinal fusion in the case of injury, correction of cutaneous scars, or deep facial wrinkles (Mandal et al., 2020).

Recently, formulations named Neo-Kidney Augment™ (NKA) involve kidney autologous cells in a gelatin hydrogel intended to deliver the same in the renal cortex region is under investigation. Therapy is being evaluated to delay renal replacement in diabetic patients and currently it is in clinical phase II (https://clinicaltrials.gov/ct2/show/NCT03270956)^1^ Another gelatin-based hydrogel is being evaluated for the delivery of autologous human cardiac-derived stem cells in patients of ischemic cardiomyopathy. The formulation is currently in the first phase of a clinical trial (https://clinicaltrials.gov/ct2/show/NCT00981006)^2^.

### Future Prospects, Patents, and Commercial Status of Protein Nanohydrogels

Nanogels are considered promising biomaterials for drug delivery systems, however more elaborated studies are needed at the single-cell level. Investigations are needed for identifying the mechanisms of uptake of nanogels, especially at the blood-brain barrier. In addition to this, pharmacokinetic properties, toxicity, and stability studies also need more research to utilize them as drug delivery systems. Although a good number of research studies focused on protein nanohydrogels, only a limited number of patents are filed in this area (Table 2), the probable reason may be the associated challenges in the development and commercialization of such delivery systems. Patents were searched from the Google patents and WIPO database.

**TABLE 2:** Patents filed on protein-based nanohydrogels.

Sr no.	Year of filing	Patent no.	Description of patent	Protein involved	Inventors
1	2020	KR20200023613A	Nanohydrogel for filler procedure with a three-dimension network structure using exosome surface protein and use thereof	Exosome surface protein	S. H. Seon, J. B. Geun
2	2019	CN109316440 A	Temperature-sensitive liquid crystal nanohydrogel, and its preparation method and application as controlled release drug carrier and in preparing *in situ* injection and interventional embolic agent	Egg yolk lecithin	L. Liang, H. Liping, Z. Yi, Z. Yiyi, M. Fanling
3	2011	WO2011070529 A2	Dextrin hydrogel for biomedical applications	Collagen and fibronectin	P.D.A Gama, F. Miguel, M. Molinos, M. Cabral
4	2013	CN103351470B	Elastin hydrogel and preparation method thereof	Elastin	H. Xin, Y. Yufang, S. Yuezai
5	2020	CN111375357 A	Method for preparing amphiphilic multifunctional nano-aerogel	Gelatin	B. Zhishan, T.T. Wang and B.Z. Shenghao
6	2019	CN109867801 A	Preparation method of magnetic nanohydrogel	Gelatin	H. Jianghong, H. Z.Wang, D. Xiong
7.	2005	WO2005042048A2	Bioactive hydrogel compositions for regenerating connective tissue	Gelatin	R.S. Hill, R.C. Klann, F.V. Lamberti
8	2001	US20020091165A1	Carboxyl-modified superabsorbent protein hydrogel	Fish protein isolate	S. Damodaran
9	2014	US8835395B2	Protein peptide hydrogels	Peptides of 30 amino acid residues	X. Susan, S.H. Huang
10	2004	US6821331B2	Protein-polysaccharide hybrid hydrogels	Soy bean protein isolate and fish protein isolate	S. Damodaran

There are many challenges which need to be addressed in order to create commercial potential for protein nanohydrogel-based drug delivery systems. Certain factors which limit the translation of a protein nanohydrogel from the laboratory to clinical practice are mainly relevant to its high cost, complex preparation methodology, storage complications, stability issues, regulatory complexity, and requirement of sophisticated analytical techniques. A protein nanohydrogel generally contains proteins from natural origin with high water content which make them susceptible to microbial contamination and limits sterilization options. Moreover, upon storage, NGs may lose water content which may alter their properties, so storage conditions for NGs also have to be established by extensive research. These nanogels have limitations pertaining to their inherent stability issues as proteins are sensitive toward pH, temperature, and salt concentrations. Nanogels intended to deliver bioactive entities are regulated as a combination product, therefore, require complex regulatory approval which further reduces their commercial viability (Wurm et al., 2014; Spiller and Vunjak-Novakovic, 2015; Li and Mooney et al., 2016).

## Conclusion

Applications of protein nanohydrogels are being explored in diverse disciplines including delivery of bioactive compounds. Properties of nanohydrogels depend upon certain factors such as the nature of constituent protein, other conjugated polymeric systems, temperature, pH, type of ionic system as well as ionic strength, oxidative-redox environment, and other reagents used during the gelation process. By tactfully tuning the above factors, the release profile of bioactive compounds from the hydrogel matrix can be adjusted.

Their nano size facilitates transport within systemic circulation while their amphiphilic nature enables incorporation of hydrophobic as well as hydrophilic drugs, including charged species.

Properties and size of nanohydrogels can be customized to avoid clearance by phagocytic cells and can reach the target by an active as well as passive route. Protein nanogels, especially those with self-assembly properties, are being explored as versatile vehicles in vaccine delivery to produce a robust immune cell response. Protein hydrogels can protect incorporated cells, peptides, biomolecules, and drugs from the harsh environment. Proteins or peptides can easily undergo self-assembly in the absence of external binding agents via an internal non-bonding interaction.

Although significant work has been done in preparation strategies and application domains of nanohydrogels, however, translating them into success clinical vehicles remains a challenge. A significant number of studies confirmed protein nanogels as good delivery vehicles for nutraceuticals, small molecules, and macromolecules, however findings are mainly limited to the *in vitro* level with very limited pre-clinical or clinical reports. In order to translate them into successful clinical delivery vehicles, more investigations are required on their pharmacokinetics behavior, biodistribution, toxicity studies, and mutual interactions between nanogels and physiological environments. Moreover, to facilitate commercialization, setting of regulatory issues, therapeutic dosage identification, pharmacodynamics characterization, optimization of storage conditions, robust characterization techniques, and uniformity in the formulation etc., these crucial factors need to be considered.

## Footnotes

1
https://clinicaltrials.gov/ct2/show/NCT03270956

2
https://clinicaltrials.gov/ct2/show/NCT00981006
